# Impact of ivermectin mass drug administration on burden of soil-transmitted helminths in onchocerciasis control and elimination programs, Yeki district, southwest Ethiopia

**DOI:** 10.1371/journal.pone.0263625

**Published:** 2022-02-10

**Authors:** Gebremedhin Gebrezgabiher, Delenasaw Yewhalaw, Mio Ayana, Asrat Hailu, Zeleke Mekonnen

**Affiliations:** 1 School of Medical Laboratory Sciences, Institute of Health, Jimma University, Jimma, Ethiopia; 2 College of Veterinary Medicine, Samara University, Samara, Ethiopia; 3 Tropical and Infectious Diseases Research Center, Jimma University, Jimma, Ethiopia; 4 Department of Microbiology, Immunology, and Parasitology, School of Medicine, College of Health Sciences, Addis Ababa University, Addis Ababa, Ethiopia; Iran University of Medical Sciences, ISLAMIC REPUBLIC OF IRAN

## Abstract

Community-directed treatment with ivermectin (CDTi) is the primary strategy employed to control and eliminate human onchocerciasis in Ethiopia. After long-term mass distribution for onchocerciasis, ivermectin is expected to have additional benefits beyond the envisioned targets by reducing the burden of other co-endemic parasitic infections as to STHs. To date, studies focused on the collateral impact of CDTi on STH in Ethiopia are scanty. Two community-based cross-sectional surveys (baseline in 1997 and post-CDTi in 2017) were conducted to evaluate the impact of long-term CDTi employed to control and eliminate onchocerciasis on the burden of STH infections in Yeki district of southwest Ethiopia. Stool samples were collected and examined using Ritchie`s concentration and Kato-Katz techniques in the baseline and current study, respectively. Overall, 188(38.3%, 95% Confidence interval (CI) 34.1–42.7%) individuals were positive at least for any of the STH species from 491 study participants in the post-CDTi. The prevalence of *A*. *lumbricoides*, hookworms, and *T*. *trichiura* was 11.2% (95% CI 8.7–14.3%), 16.3% (95% CI 13.3–19.8%), and 29.9% (95% CI 26.1–34.1%), respectively. Individuals aged 5–9 years had a significantly higher prevalence of *A*. *lumbricoides* (Adjusted odds ratio (AOR) 6.5, 95% CI 1.7–25.4), *T*. *trichuria* (AOR 8, 95% CI 2.6–25.1), and any STH infection (AOR 5, 95% CI 1.7–14.7) than those of ≥ 51 years. Also, significantly higher prevalences of *T*. *trichuria* infection were observed in individuals aged 10–14 years (AOR 4.1, 95% CI 1.7–9.9), 15–20 years (AOR 3.1, 95% CI 1.2–8.1), 21–30 years (AOR 2.4, 95% CI 1.1–5.5), and 31–40 years (AOR 3.2, 95% CI 1.3–7.5) compared with those of ≥ 51 years. The prevalence of *A*. *lumbricoides* was significantly higher in males (AOR 0.5, 95% CI 0.3–0.9). Of the 491 study participants, only data from 400 individuals who had not been involved in a mass drug administration (MDA) with other STH anthelmintics were considered in the comparative analysis. Before CDTi, the prevalence of *A*. *lumbricoides*, *T*. *trichiura*, hookworm, and any STH infection was 47.1% (95% CI 41.6–52.7%), 3.3% (95% CI 1.8–5.9%), 37.9% (95% CI 32.7–43.5%), and 58.8% (95% CI 53.2–64.1%), respectively. Long-term CDTi considerably reduced the prevalences of *A*. *lumbricoides* and hookworm by 76.2% and 56.9%, respectively (p < 0.001). Nonetheless, CDTi did not affect the prevalence of *T*. *trichiura* infection and, in contrast, it was significantly higher in the current study (P < 0.001). Overall post-CDTi prevalence of any STH infection was considerably lower than reported in the baseline (p < 0.001). It is evidenced that long-term CDTi for onchocerciasis control and elimination had additional benefits by reducing the prevalence of STH infections specifically of *A*. *lumbricoides* and hookworm, but had no impact on infections with *T*. *trichuria*. Our finding of additional health benefits of large-scale ivermectin administration taking it will aid to increase positive engagement and sustain participation of communities during MDA campaigns, and strengthen governmental and non-governmental organizations (NGOs) support for the undergoing national onchocerciasis elimination program.

## Introduction

Soil-transmitted helminth (STH) infections, caused by a group of parasitic nematode worms commonly known as roundworms (*Ascaris lumbricoides*), whipworms (*Trichuris trichiura*), and hookworms (*Ancylostoma duodenale* and *Necator americanus*), is the most common type of infection worldwide [1]. An estimated 5.3 billion people are at risk, while 1.5 billion are infected with at least one of the STH species [2]. The most significant number of infection cases occur in tropical and sub-tropical regions of the developing world where adequate supply of water and sanitation is lacking, mainly in rural areas of sub-Sahara Africa, the Americas, Southeast Asia, and China [3]. The morbidity of STH is directly linked to the number of worms harboured by infected individuals [4]. The higher the number of worms in an infected person, the more it causes severe morbidity [5], including abdominal pain, diarrhea, blood and protein loss, rectal prolapse, and physical and cognitive growth retardation particularly in school-aged children (SAC) [6]. In 2017, the global burden of infections with STH was estimated to be 1.9 million disability-adjusted life years (DALYs) [7].

Ethiopia is one of the countries in sub-Sahara Africa where the enormous burden of STH infections is found [8]. STH infections are distributed widely throughout the country [9] with varied prevalence rates between geographical areas [10]. The nationwide mapping of STH and schistosomiasis conducted in 2014 and 2015 demonstrated that out of 833 country’s districts 741 are endemic for STHs [9, 11]. It is estimated that 79 million people live in STH endemic areas, which comprised 9.1 million pre-school-aged children, 25.3 million SAC, and 44.6 million adults [9, 11]. STHs are responsible for significant morbidity, for example, STHs caused an estimated loss of 82.7 thousand DALYs in 2015, representing 14.3% of the total DALYs lost because of all Neglected tropical diseases (NTDs) [12].

Ivermectin also known as Mectizan®, a product of Merck & Co, Inc., is a drug of choice to control human onchocerciasis [13], a skin disfiguring and blinding disease caused by infection with the filarial worm *Onchocerca volvulus* that affected millions of people in tropical areas [14]. Since registered for human use against onchocerciasis in 1987 the manufacturer Merck & Co Inc., donated free of charge [15] to any government or non-governmental organization (NGO) involved in onchocerciasis control. Accordingly, it has been extensively used in mass drug administration (MDA) to control the disease across Africa, where over 99% of all the onchocerciasis cases in the world were historically documented [16, 17]. Yet, it is the only drug being in use in the current World Health Organization (WHO) AFRO Region Expanded Special Project for the Elimination of Neglected Tropical Diseases (ESPEN) program for onchocerciasis elimination [18]. The primary effects of ivermectin are against skin microfilariae [19, 20]. It is a potent microfilaricidal and partially embryostatic agent after frequent application [21–23]. In fact, it is a broad-spectrum anti-helminthic drug that also impacts other co-endemic parasitic infections [14, 24]. Several authors in different parts of the world reported the impact of ivermectin against STHs [5, 25–32].

Community-wide mass ivermectin treatment (also referred to as Community-directed treatment with ivermectin: CDTi) is the primary strategy for controlling and eliminating onchocerciasis in Ethiopia. The program was launched in 2001 in 16 highly endemic districts of Kaffa-Sheka zone in South Nations Nationalities Peoples Region (SNNPR) [9, 33], later expanded to other areas and continued to the present day as part of the national efforts to interrupt transmission of *O*. *volvulus* infection [34].

An epidemiological survey carried out before the launch of CDTi in communities of Yeki revealed that STHs were highly endemic and found co-morbid with onchocerciasis [35]. We hypothesized that the ongoing mass ivermectin treatment for onchocerciasis could have an additional benefit/collateral impact to the communities taking the drug by preventing or reducing part of the burden related to STH infections. These additional benefits of ivermectin are being used to sensitize communities to participate in CDTi campaigns. To date, studies focused on the collateral impact of CDTi on STH in Ethiopia are scanty, except Taticheff and his colleagues [36] that attempted to investigate the effect of repeated ivermectin treatment in Bebeka coffee plantation in southwest Ethiopia during the early period of CDTi. The ancillary health impact of long-term mass ivermectin treatment has not been quantitatively measured till now, and its impact remains unknown. It is of interest to know whether the population living in communities of onchocerciasis endemic areas are being benefited from the ongoing long-term CDTi program by reducing the prevalence of STH infections. Therefore, this study was conducted to estimate the burden of STH and assess the impact of the long-term CDTi program in reducing the prevalence of STH in communities of Yeki district of SNNPR of southwest Ethiopia compared to STH prevalence data collected before the launch of the program.

## Materials and methods

### Study setting

**Fig 1:**
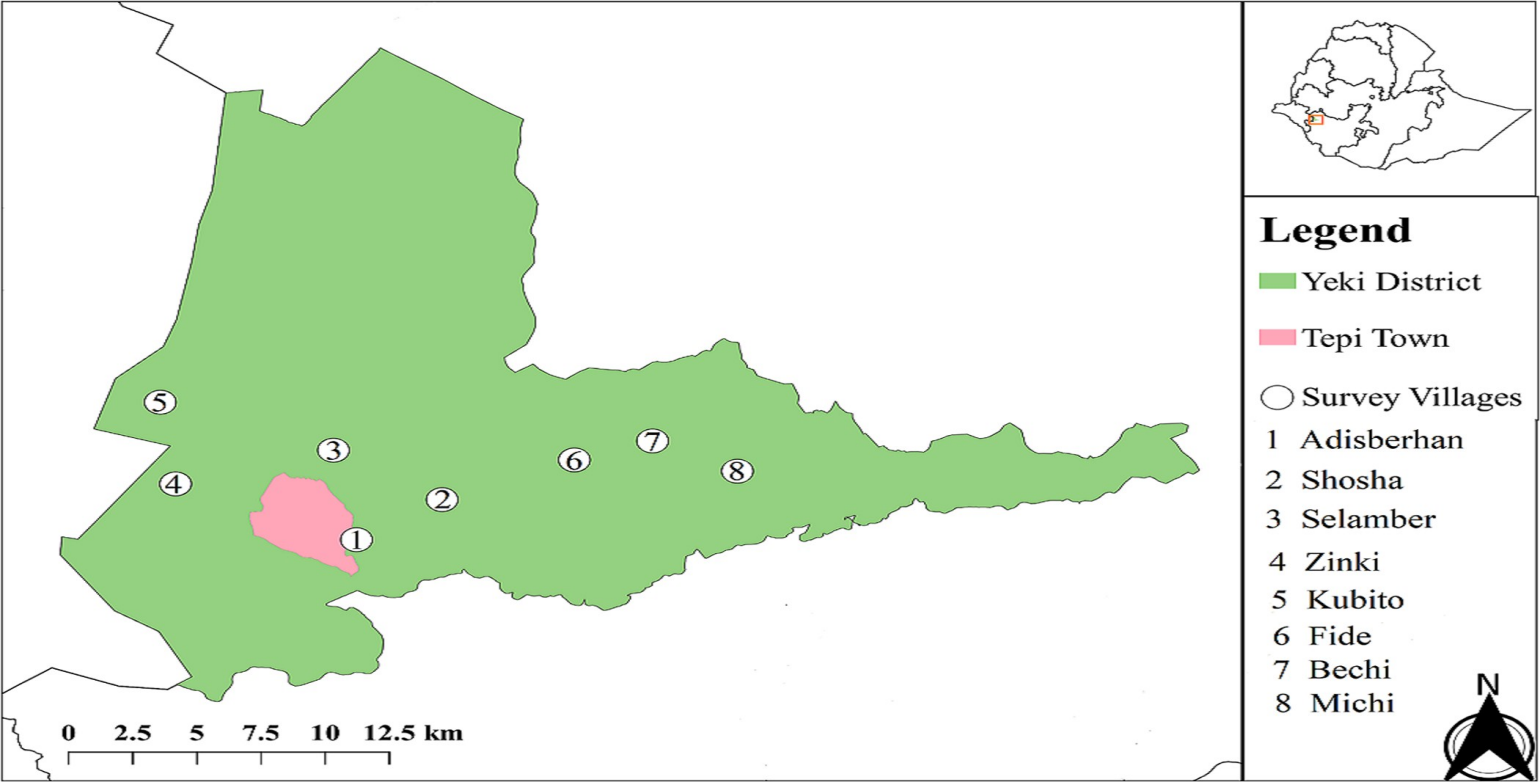
Study map showing study *kebeles* (the smallest administrative unit) in Yeki district of SNNPR, southwest Ethiopia. QGIS version 3.10, a free and open source GIS software, was used to generate the map of the study area [38]. The spatial data referring to the study sites are acquired using a handheld GPS device (Garmin’s GPSMAP 60CSx, Garmin International Inc., Olathe, Kansas, USA) during sample collection. The administrative spatial data (Open Street Map shapefile) used to prepare this study map was downloaded from GADM (https://gadm.org/download_country_v3.html), which is free and open source database of global administrative areas.

Two community-based cross-sectional surveys were carried out in communities of Yeki district of SNNPR of southwest Ethiopia. The baseline survey before the launch of ivermectin distribution for onchocerciasis control and the second after more than 15 years of CDTi in similar communities of the study district. Yeki is located at 7°14’60’’ latitude, 35°24’60’’ longitude, and an altitude of 1200 meters above sea level representing a low altitude. Characterized by hot and humid climate, Yeki district has an average annual rainfall of 1700 millimeters and a monthly temperature ranging from a minimum of 14.7–17.7°C to a maximum of 26.2–31.4°C. A detailed geographic description of the study area is available elsewhere [37]. The CDTi program launched in Yeki district in 2001 and was administered annually until 2012. In 2013, the program shifted to biannual MDA as part of the efforts to achieve the elimination of onchocerciasis. Yeki district was selected for this study because STH prevalence data were collected before the launch of CDTi for control of onchocerciasis to be used as baseline data. Moreover, STH control program is at its early stage of implementation in Yeki district of SNNPR. The program was launched in February 2016 as a biannual MDA campaign targeting SAC. Before this study, only three rounds of MDA were conducted (two rounds in 2016 and one in 2017). It is delivered through school and community-based deworming program. Adisberhan *kebele* based on the presence of pre-CDTi STH prevalence data and seven other nearby *kebeles* with similar ecology, and socio-economic activities (Bechi, Fide, Kubito, Michi, Shosha, Selamber, and Zinki) were purposively selected for this study (Fig 1).

### Surveys and sample processing

In May 1997, the first survey was carried out in a population aged 10 years old and above (n = 308), who lived in communities of Yeki district at least for five years [35]. Community members aged ten years and above were informed through community leaders and health workers to come to a village clinic. The purpose of the study was explained and asked to voluntarily participate in the study. After obtaining a verbal consent from each study participant (parents or guardians in case of children), slightly more than one gram of freshly excreted stool sample was collected and preserved in 10% formalin in screw-capped vials. The samples were processed by Ritchie’s concentration technique, and the concentrate was examined under a microscope for the presence of parasite eggs [35]. The detailed laboratory procedure is available elsewhere [39].

From the third week of October to the end of November 2017, another cross-sectional survey was carried out in the area where the previous survey was performed. Stool samples were collected during a CDTi coverage verification survey in the district, which included the *kebeles* in this study. This study used the same household sampling technique as the CDTi coverage survey. Briefly, a systematic sampling of households was carried out, with the sampling interval determined by dividing the total number of households in each *kebele* by the sample size assigned to each *kebele*. After the purpose of the study was explained to them, household members who volunteered to participate in the study gave their voluntary consent and a face-to-face interview was done to obtain socio-demographic data (sex, age, religion, and ethnicity) and their ivermectin treatment status in the last round of the CDTi campaign in 2017. Next, a clean plastic screw-cup container pre-labeled with the individual’s code was provided to all voluntary participants. The participant was instructed to scoop a thumb-sized stool sample using a provided scoop into the container. Parents and guardians were instructed to guide their children during the sample collection to ensure that children placed their stool samples into the container. The next morning, the containers with stool samples were collected and placed into a zip-locked plastic bag. Stool samples were examined with the Kato-Katz technique [40], a WHO-recommended diagnostic method for identifying and quantifying STH eggs in stools [41], which consist of a single thick smear technique with a 41.7mg template that allows a standardized amount of stool to be examined and eggs to be counted [42]. The samples were examined by a qualified laboratory technician within one hour after slides’ preparation to ensure that all STH species potentially present in the preparation should be identified. All eggs found in the preparations were identified and counted and the results were expressed as eggs per gram (EPG) of stool to express the intensity of the infection.

### Ethical consideration

The study obtained ethical approval from the Institutional Review Board of the Institute of Health Jimma University (RPGC/170/06). The aim of the study was explained to the study participants, and they were informed that they are free to participate. Informed written consent was obtained for those age 18 and above, or from parents or guardians for participants less than 18 years. Permission was obtained from the Zonal Health Bureau, District Health Bureau, and *Kebele* Authorities. The collected information was kept confidential in compliance with established Human Subject Protection guidelines.

### Statistical analysis

All data were checked, cleared, and entered in Microsoft Excel Sheet (2013 Microsoft Corporation). Statistical analyses were performed using RStudio version 3.2.3 software. Descriptive statistics were used to summarize the socio-demographic characteristics of study participants. Prevalence of STH infection was expressed as the mean proportion of individuals testing positive out of total examined with a 95% confidence interval (CI). The quantity of STH eggs was counted and calculated following WHO [43], and the infection intensity of each species was stratified into three categories (light, moderate, and heavy) [43]. The prevalence of STH infections was determined following disaggregated by sex and age group. Multivariate logistic regression analysis was used to calculate the odds ratio and the 95% CI to observe the risk associations between STH infections and the variables used. The difference in the prevalence of STH infections between baseline and post-CDTi studies was calculated, means were compared using the Chi-Square test and p-value ≤ 0.05 was considered significant during the analysis.

## Results

### Socio-demographic characteristics of study participants

Of 512 study participants who agreed to participate, 495 (96.7%) provided stool samples. The samples from four participants were not adequate and were not processed. Stool samples from 491individuals were processed. Of these, 55.4% (272/491) were females. The study participants’ age ranged from 5–80 years. It was shown that 30.5% (150/491) of the study participants were in the age range of 21–40 years. As per interviewees’ declarations, 90.6% (445/491) of the study participants reported they took ivermectin in the last rounds of the CDTi campaign in 2017. Table 1 shows the socio-demographic characteristics of the study participants.

**Table 1:** Socio-demographic characteristics of the study participants from Yeki district of southwest Ethiopia (2017).

Variable		Frequency	Percent
**Sex**	Female	272	55.4
	Male	219	44.6
**Age (years)**	5–9	21	4.3
	10–14	67	13.6
	15–20	47	9.6
	21–30	150	30.5
	31–40	90	18.3
	41–50	60	12.2
	≥ 51	56	11.4
**Religion**	Protestant Christian	246	50.1
	Orthodox Christian	146	29.7
	Muslim	99	20.2
**Ethnicity**	Amhara	178	36.3
	Kafficho	64	13
	Majang	86	17.5
	Manja	70	14.3
	Sheko	36	7.3
	Bench	26	5.3
	Oromo	21	4.3
	Shakicho	10	2
**Ivermectin treatment in May 2017 CDTi**	No	46	9.4
	Yes	445	90.6

CDTi: Community-directed Treatment with Ivermectin.

### Trend of reported CDTi coverage

**Fig 2:**
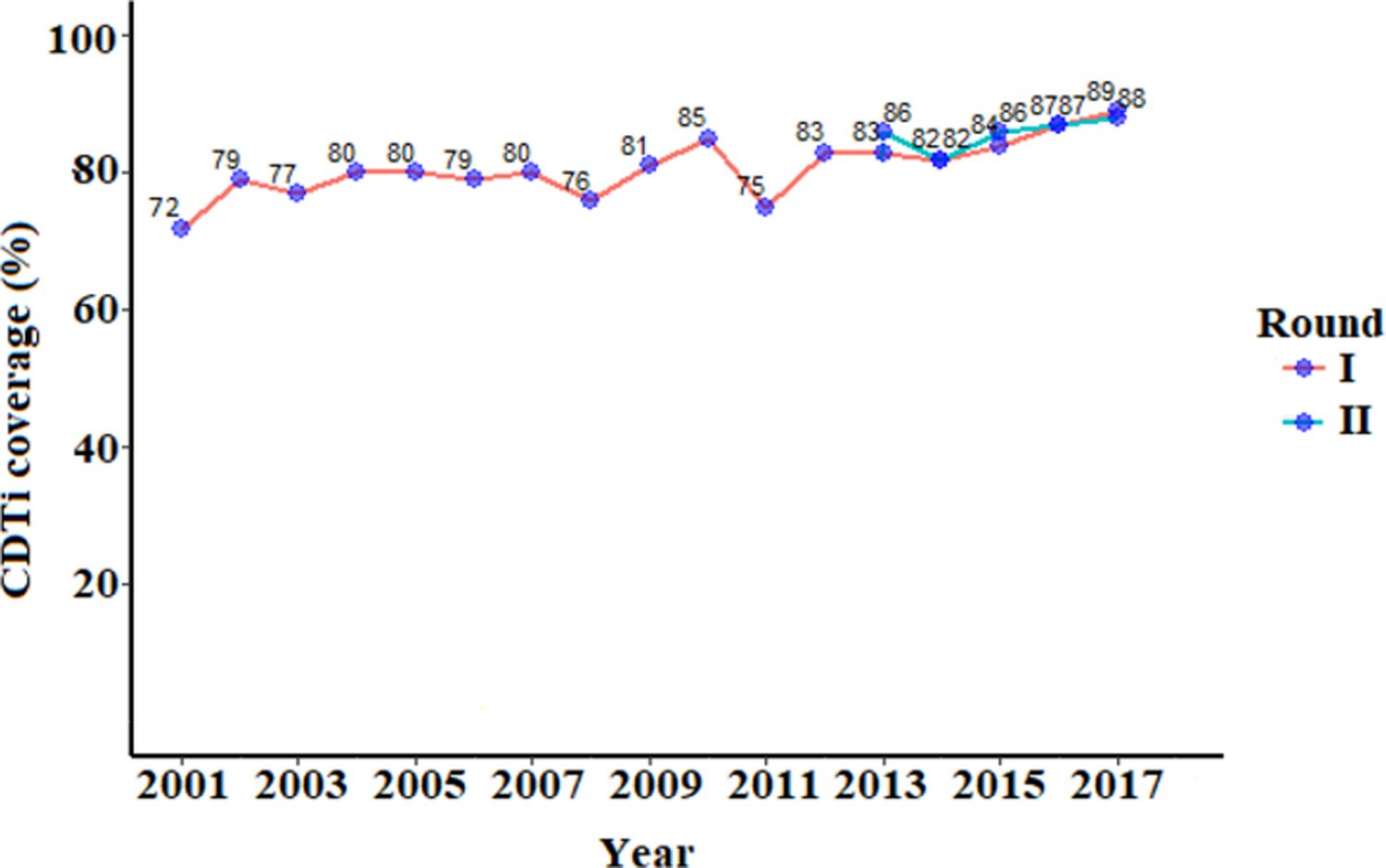
Trend of CDTi coverage rate in Yeki district of SNNPR, southwest Ethiopia (2001–2017). (Source: Yeki district health office).

Community drug distributors (health extension workers) report CDTi coverage rates to the district health office following every ivermectin distribution campaign. Consecutive registers of community drug distributors show high CDTi coverage rates (above 80%, which is the minimum threshold for the elimination of onchocerciasis) were achieved in most of the MDA rounds in Yeki district of SNNPR. Fig 2 depicts the trend of reported CDTi coverage rate in Yeki District since the launch of the program in 2001.

### Prevalence and intensity of STH infections

Overall, 188(38.3%, 95% CI: 34.1–42.7%) individuals were at least positive for any of STH species (Table 2). The presence of three species of STH: *A*. *lumbricoides*, hookworm and *T*. *trichiura* was 55(11.2%, 95% CI: 8.7–14.3%), 80(16.3%, 95% CI: 13.3–19.8%), and 147 (29.9%, 95% CI: 26.1–34.1%), respectively. Moreover, 112 (22.8%, 95% CI: 19.3–26.7%) and 76 (15.5%, 95% CI: 12.6–18.9%) of the infected individuals had single and mixed infections, respectively. Among the study participants with mixed infections, double and triple STH infections were detected in 58(3.4%; 95% CI: 2.3–5.7%) and 18(3.7%; 95% CI: 2.3–5.7%), respectively (Table 2).

**Table 2:** STH infection in Yeki district of southwest Ethiopia (2017).

Infection status	Type of parasite	Frequency	Percent (95% CI)
**Single infection**	*A*. *lumbricoides*	9	1.8(1, 3.5)
	*T*. *trichiura*	78	16(12.9, 19.4)
	Hookworm	25	5.1(3.8, 7.4)
**Double infection**	*A*. *lumbricoides* and *T*. *trichiura*	21	4.3(2.8, 6.5)
	*A*. *lumbricoides* and hookworm	7	1.4(0.7, 2.9)
	*T*. *trichiura and* hookworm	30	6.1(4.3, 8.6)
**Triple infection**	*A*. *lumbricoides*, *T*. *trichiura* and hookworm	18	3.7(2.3, 5.7)
**Overall STH infection**		188	38.3 (34.1, 42.7)

CI: Confidence Interval; STH; Soil-transmitted helminths; *A*. *lumbricoides*: *Ascaris lumbricoides*; *T*. *trichiura*: *Trichuris trichiura*.

Results of multivariate logistic regression analysis showed that study participants aged 5–9 years had a significantly higher prevalence of *A*. *lumbricoides* (Adjusted odds ratio (AOR) 6.5, 95% CI 1.7–25.4, p = 0.007), *T*. *trichuria* (AOR 8, 95% CI 2.6–25.1, p<0.001), and any STH infection (AOR 5, 95% CI 1.7–14.7, p = 0.003) than the older counterparts (≥ 51 years). Similarly, a significantly higher prevalence of *T*. *trichuria* infection was observed in individuals in the age ranges of 10–14 years (AOR 4.1, 95% CI 1.7–9.9, p = 0.002), 15–20 years (AOR 3.1, 95% CI 1.2–8.1, p = 0.021), 21–30 years (AOR 2.4, 95% CI 1.1–5.5, p = 0.037), and 31–40 years (AOR 3.2, 95% CI 1.3–7.5, p = 0.009) compared with ≥ 51 years old. There was no significant association between sex and infection rates of either specific species of STH or any STH infection, except *A*. *lumbricoides*, where the prevalence of infection was significantly lower in females than in males (AOR 0.5, 95% CI 0.3–0.9, p = 0.022) (Table 3).

**Table 3:** Prevalence of species-specific STH infection by sex and age in Yeki district of southwest Ethiopia (2017).

Parasite		Sex		Age (years)						
		Female (n = 272)	Male (n = 219)	5–9	10–14	15–20	21–30	31–40	41–50	≥ 51
***A*. *lumbricoides***	% positive	22(40)	33(60)	7(33.3)	5(7.5)	2(4.3)	19(12.7)	15(16.7)	3(5)	4(7.1)
	AOR (95% CI)	0.5(0.3–0.9)	1	6.5(1.7–25.4)	1.1 (0.3–4.1)	0.6(0.1–3.3)	1.9(0.6–5.8)	2.6(0.8–8.3)	0.7(0.2–3.2)	1
	P-value	0.022^†^		0.007^†^	0.946	0.537	0.269	0.106	0.630	
***T*. *trichiura***	% positive	84(57.1)	63(42.9)	12(57.1)	27(40.3)	16(34)	43(28.7)	31(34.4)	10(16.7)	8(14.3)
	AOR (95% CI)	1.1 (0.7–1.7)	1	8(2.6–25.1)	4.1(1.7–9.9)	3.1(1.2–8.1)	2.4(1.1–5.5)	3.2(1.3–7.5)	1.2(0.4–3.3)	1
	P-value	0.593		<0.001^†^	0.002^†^	0.021^†^	0.037^†^	0.009^†^	0.724	
**Hookworm**	% positive	42(52.5)	38(47.7)	5(23.8)	13(19.4)	8(17)	25(16.7)	17(18.9)	4(6.7)	8(14.3)
	AOR (95% CI)	0.9 (0.5–1.4)	1	1.9(0.6–6.6)	1.4(0.6–3.8)	1.2(0.4–3.6)	1.2(0.5–2.8)	1.4(0.6–3.5)	0.4(0.1–1.5)	1
	P-value	0.596		0.325	0.454	0.703	0.679	0.474	0.188	
**Any STH**	% positive	105(38.6)	83(37.9)	14(66.7)	29(43.3)	20(42.6)	57(38)	39(43.3)	13(21.7)	16(28.6)
	AOR (95% CI)	1.1(0.7–1.6)	1	5(1.7–14.7)	1.9(0.9–4.1)	1.9(0.8–4.2)	1.5(0.8–2.9)	1.9(0.9–3.9)	0.7(0.3–1.6)	1
	P-value	0.745		0.003^†^	0.093	0.14	0.21	0.075	0.392	

AOR: Adjusted odds ratio; CI: Confidence Interval; STH: Soil-transmitted helminth; ^†^: Significant at p< 0.05.

Most of the study participants (79.8%, 95%CI 73.5–84.9%) had light STH infection, while the remaining 38(20.2%, 95%CI 15.7–27.5%) had a moderate infection. Light intensity of infection was detected in 50(90.9%, 95%CI 80.4–96.1%), 120(81.6%, 95%CI 74.6–87.1%), and 68(85%, 95%CI 75.6–91.2%) for *A*. *lumbricoides*, *T*. *trichuria*, and hookworms, respectively, while moderate infection intensity for *A*. *lumbricoides*, *T*. *trichuria*, and hookworm was observed in 5(9.1%,95%CI 3.9–19.6%), 27(18.4%, 95%CI 12.9–25.4%) and 12(15%, 95%CI 8.8–24.4%), respectively. None was infected with heavy infection.

### Impact of CDTi on prevalences of STH infections

Table 4 compares the baseline and post-CDTi STH prevalence data based on the age of the study participants. SAC are involved in the recently launched MDA program against STH in Yeki. For comparison of the current prevalence rates of any helminth infection with the baseline prevalence, study participants younger than 15 years were excluded and only data from 400 individuals were considered for analysis. Before the launch of CDTi, the overall prevalence of any STHs was 58.8% (95% CI 53.2–64.1%) (181/308) [35]. Overall, a decline in the prevalence of any STH infection was observed in the current study compared to the baseline within all age groups, however, the decline in the prevalence of any STH infection was significant only in the age group of 21–30 years (p = 0.03).

**Table 4:** Baseline and post-CDTi prevalence of any STH infection disaggregated by age in Yeki district of southwest Ethiopia.

Variable		Baseline		Post-CDTi		% Change	χ^2^ (p-value)
		N examined	n positive (%)	N examined	n positive (%)		
**Age (years)**	11–20^±^	55	38(69.1)	44	20(45.5)	34.2↓	1.12(0.29)
	21–30	146	88(60.3)	150	57(38)	37↓	4.6(0.03^†^)
	31–40	51	29(56.8)	90	39(43.3)	23.8↓	0.56(0.45)
	41–50	24	8(33.3)	60	13(21.7)	34.8↓	0.34(0.56)
	≥ 51	32	18(56.3)	56	16(28.6)	49.2↓	2.14(0.14)

STHs; Soil-transmitted helminths; ↓:% reduction in the prevalence of infection; ^†^:statistically significant; ^±^: only study participants aged 16–20 years were included for the post-CDTi study for comparison with the 11–20 years old from the baseline study.

**Fig 3:**
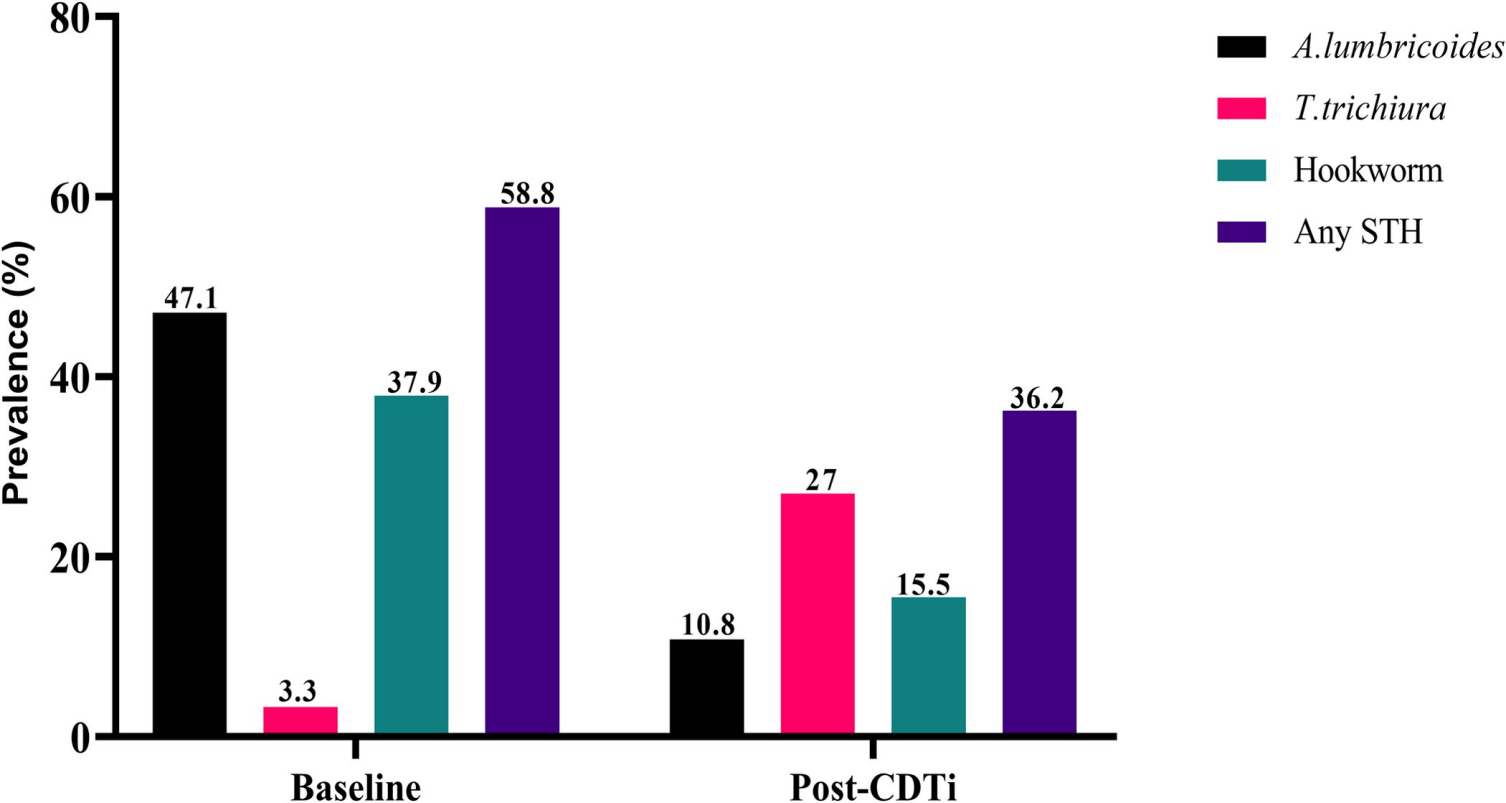
Comparison of baseline and post-CDTi data of prevalence of STH in Yeki district of SNNPR, southwest Ethiopia.

Before CDTi, 145(47.1%, 95% CI 41.6–52.7%), 10(3.3%, 95% CI 1.8–5.9%), and 117(37.9%, 95% CI 32.7–43.5%) individuals had *A*. *lumbricoides*, *T*. *trichiura*, and hookworms, respectively [35]. Long-term CDTi considerably reduced the prevalences of *A*. *lumbricoides* and hookworm infections by 76.2% and 56.9%, respectively (p < 0.001). However, one most interesting observation was the CDTi did not appear to reduce the prevalence of *T*. *trichiura* infection and, in contrast, it was significantly higher in the current study (p < 0.001). Overall, the prevalence of any STH infection was considerably lower than reported in the baseline (p < 0.001). Fig 3 shows the baseline and post-CDTi prevalences of STHs in the study district.

## Discussion

STH infection is the most common and persistent parasitic infection worldwide mainly in tropical and sub-tropical areas of the developing world where adequate water supply and sanitation are lacking [3]. Ethiopia is among the countries in sub-Sahara Africa having the highest burden of STH [44] and have enormous consequences on the health and educational performance of SAC [9]. The WHO`s STH control strategy emphasizes controlling STH morbidity through periodic MDA with a single dose of albendazole or mebendazole to the population at risk of infection [45]. The frequency of MDA is based on the overall prevalence of STH, annual when the prevalence is 20–50%, and biannual when the prevalence is above 50% [46].

The success of the onchocerciasis control and elimination program is evaluated by its impact on *O*. *volvulus* prevalence and intensity. In Yeki district of SNNPR, where CDTi has been running from 2001–2017, the program has noticeably reduced *O*. *volvulus* prevalence to zero [37]. Data collected before the launch of CDTi from this area showed that STH were highly endemic and many individuals were detected concomitantly infected with both STH and onchocerciasis [35]. After the long-term mass ivermectin distribution for onchocerciasis, the health benefits of CDTi beyond the envisioned targets for example to STH can be confirmed. Thus, evaluating the collateral public benefits of CDTi in reducing the prevalence of co-endemic STH infections is crucial for implementing further interventions and investments from donor organizations and strengthening governmental and community support for the undergoing national onchocerciasis elimination program.

The present study revealed the prevalence of *A*. *lumbricoides*, *T*. *trichiura*, and hookworm infections were 11.2%, 29.9%, and 16.3%, respectively. The overall prevalence of any STH infection was 38.3%, indicating that STH infection is still a public health problem in the study area. This was considerably lower than findings reported from nearby areas where annual/biannual CDTi is also being implemented [47, 48]. We observed a significantly higher prevalence of *A*. *lumbricoides*, *T*. *trichiura*, and any STH infection among individuals of age range 5–9 years compared with infection in study participants aged 51 years and above. Commonly, children (especially SAC) are at high risk of STH infections [43]. Moreover, a significantly higher proportion of adults in the study district harbour *T*. *trichiura* infection, therefore, might be contributing to ongoing transmission of infection in the area. In agreement with previous findings [32, 49], we did not observe a significant difference in any STH infection between males and females. This could be because both males and females might have similar exposure to STH [32, 49].

Our study documented that long-term CDTi is significantly associated with a decreased prevalence of *A*. *lumbricoides* and hookworm infections. An earlier study that evaluated the long-term impact of annual CDTi for onchocerciasis on the prevalence of STH infections in pre-school-aged children and SAC in Imo state of eastern Nigeria also reported the same findings indicating that the prevalence of *A*. *lumbricoides* was significantly lower in children living in areas treated with 13 years of annual ivermectin treatment than children living in untreated areas [31]. In another similar study that assessed the prevalences of STH in three communities in northern Nigeria with contrasting treatment history (ivermectin only, ivermectin plus albendazole, and no MDA history) showed the lowest prevalence of each species of STH in the communities treated only with ivermectin for the control and elimination of onchocerciasis in northern Nigeria [32]. The most unusual finding of their study was the lowest prevalences of STH species in communities treated with ivermectin only even compared to the areas treated with a combination of ivermectin and albendazole [32]. Moreover, our results are consistent with previous follow-up studies that investigated the effect of repeated treatments with ivermectin against STH and shown the reduced prevalence of *A*. *lumbricoides* infection [27–29, 36, 50]. This finding also supports evidence from observations of previous field-based studies and community trials [25, 26, 51–53] that reported a reduction in the prevalence of *A*. *lumbricoides* after single-dose ivermectin treatment. Meanwhile, this finding is contrary to a previous study which has shown no important effect of up to 17 years of biannual mass ivermectin treatments on the prevalence of *A*. *lumbricoides* [30]. For hookworm infection, similar effects to our finding have been observed from a previous study that compared prevalence rates of STH before and after mass treatments with ivermectin in northeast Brazil [28]. In contrast, long-term periodic mass ivermectin treatment had little impact on the prevalence of hookworm infection [30, 31].

Our findings also showed that 15 years of continuous CDTi did not reduce the prevalence of *T*. *trichuria* infection. On the contrary, the prevalence of *T*. *trichuria* infection was high, and even significantly higher than the baseline prevalence reported in 2002, revealing *T*. *trichuria* is a public health problem in the area. The current evidence that long-term mass ivermectin treatment had no effect on *T*. *trichiura* complements the results of earlier studies [27, 50, 53, 54]. However, it is reported that regular mass biannual ivermectin treatment of communities for a period up to 17 years had a more significant impact on *T*. *trichuria* infection [30].

As far as we know, no study has investigated the long-term collateral impact of CDTi on STH epidemiology in Ethiopia. This is the first study that CDTi for onchocerciasis also led to significant reductions in *A*. *lumbricoides*, hookworm, and any STH infection prevalence at the population level. The strength of our study was the baseline level of STH infection in this area was obtained before initiating mass ivermectin distribution for onchocerciasis control. Thus, we were able to estimate the burden of STH and measure the impact of CDTi in the study area.

### Limitation of the study

The present study had the following limitations. First, the prevalence and intensity of infection estimates of STH were based on a single specimen. There is temporal variation in egg excretion over hours and days so that it is probable that this might affect the accuracy of the egg count and affect the estimated prevalence and intensity of infection in the area. Second, we have not measured the decline in the intensity of STH infection because there was no baseline data on the intensity of infection. Third, there is a large gap between the two studies. Thus, we cannot rule out a decline in the prevalence of infections due to other interventions like better access to water, sanitation and hygiene improvements, and health education programs that are thought to bring behavioural changes [55]. Hence, further investigation of predictive factors of the observed infections other than those included in this study is needed.

## Conclusions

We have assessed the prevalence and intensity of STH and evaluated the impact of long-term mass ivermectin administration employed to control and eliminate onchocerciasis on the epidemiology of STH infections in Yeki district of southwest Ethiopia. Moreover, we determined the prevalence and intensity of STH infection. Our study evidenced that the long-term CDTi program for onchocerciasis control and elimination had collateral benefits by reducing the burden of STH infections, specifically on *A*. *lumbricoides* and hookworm, but had no impact on *T*. *trichuria* infections. We believe that the finding of additional health benefits of large-scale ivermectin administration will aid to increase positive engagement and sustain participation of communities during MDA campaigns, and strengthen governmental and NGOs support for the undergoing national onchocerciasis elimination program.

## Supporting information

S1 Table Prevalence of any STH infection by kebele, religion and ethnicity of study participants from Yeki district of southwest Ethiopia (2017). (DOCX)

## List of abbreviations

AOR: Adjusted odds ratio
CDTi: Community-Directed Treatment Ivermectin
CI: Confidence Interval
epg: eggs per gram
ESPEN: Expanded Special Project for the Elimination of Neglected Tropical Diseases
DALYs: Disability Adjusted Life Years
MDA: Mass Drug Administration
NGO: non-governmental organization
NTDs: Neglected Tropical Diseases
OR: Odds ratio
SAC: School-aged Children
SNNPR: South Nations Nationalities Peoples Region
STH: Soil-transmitted Helminth
WHO: World Health Organization

## References

[1] Tchuenté LAT. Control of soil-transmitted helminths in sub-Saharan Africa: Diagnosis, drug efficacy concerns and challenges. Acta Trop. 2011; 120S: S4–S11. 10.1016/j.actatropica.2010.07.001 20654570

[2] Moser W, Schindler C, Keiser J. Efficacy of recommended drugs against soil-transmitted helminths: systematic review and network meta-analysis. BMJ. 2017; 358: 1–10. doi: 10.1136/bmj.j4307 28947636 PMC5611648

[3] Brooker S, Clements ACA, Bundy DAP. Global epidemiology, ecology and control of soil-transmitted helminth infections. Adv Parasitol. 2006; 62: 221–261. doi: 10.1016/S0065-308X(05)62007-6 16647972 PMC1976253

[4] BA B, Amazigo U. Onchocerciasis. In: Gyapong J, Boatin B, editors. Neglected Tropical Diseases-sub-Saharan Africa. Switzerland: Springer International Publishing; 2016. pp. 87–112.

[5] Yemeli LD, Djeunga HN, Lenou-Nanga CG, Azafack CD, Domche A, Thotchum FF, et al. Serious limitations of the current strategy to control Soil-Transmitted Helminths and added value of ivermectin mass administration: a population-based observational study in Cameroon. PLoS Negl Trop Dis. 2020; 14: 1–13. 10.1371/journal.pntd.0008794 PMC7665818 33141853

[6] Bethony J, Brooker S, Albonico M, Geiger SM, Loukas A, Diemert D, et al. Soil-transmitted helminth infections: ascariasis, trichuriasis, and hookworm. Lancet. 2006; 367: 1521–1532. doi: 10.1016/S0140-6736(06)68653-4 16679166

[7] GBD 2017 DALYs and HALE Collaborators. Global, regional, and national disability-adjusted life-years (DALYs) for 359 diseases and injuries and healthy life expectancy (HALE) for 195 countries and territories, 1990–2017: a systematic analysis for the Global Burden of Disease Study 2017. Lancet. 2018; 392: 1859–18922. doi: 10.1016/S0140-6736(18)32335-3 30415748 PMC6252083

[8] Karagiannis-Voules, Biedermann P, Ekpo UF, Garba A, Langer E, Mathieu E, et al. Spatial and temporal distribution of soil-transmitted helminth infection in sub-Saharan Africa: a systematic review and geostatistical meta-analysis. Lancet Infect Dis. 2014; 3099:71004–71007. doi: 10.1016/S1473-3099(14)71004-7 25486852

[9] Federal Ministry of Health. Second Edition of Ethiopia National Master Plan For Neglected Tropical Diseases. Addis Ababa, Ethiopia. 2016; pp. 1–78.

[10] Federal Ministry of Health. Health Sector Transformation Plan (2015/16–2019/20). Addis Ababa, Ethiopia. 2015. pp. 1–182.

[11] Negussu N, Mengistu B, Kebede B, Deribe K, Ejigu E, Tadesse G, et al. Ethiopia schistosomiasis and soil-transmitted helminths control programme: progress and prospects. Ethiop Med J. 2017; 55: 75–80. 28878432 PMC5582635

[12] Deribew A, Kebede B, Tessema GA, Adama YA, Misganaw A, Gebre T, et al. Mortality and Disability-Adjusted Life-Years (DALYs) for Common Neglected Tropical Diseases in Ethiopia, 1990–2015: Evidence from the Global Burden of Disease Study 2015. Ethiop Med J. 2017; 55(Suppl 1): 3–14. 28878427 PMC5582634

[13] Hotez PJ. Mass Drug Administration and Integrated Control for the World’s high Prevalence Neglected Tropical Diseases. Transl Med. 2009; 1–6. doi: 10.1038/clpt.2009.16 19322166

[14] Crump A. Ivermectin: enigmatic multifaceted ‘ wonder ‘ drug continues to surprise and exceed expectations. J Antibiot. 2017; 70: 495–505. 10.1038/ja.2017.11 28196978

[15] Etya’ale D. Vision 2020: Update on Onchocerciasis. *Community Eye Heal*. 2010; 14: 19–21. PMC1705929 17491908

[16] Mackenzie CD, Homeida MM, Hopkins AD, Lawrence JC. Elimination of onchocerciasis from Africa: possible? Trends Parasitol. 2012; 28: 16–22. doi: 10.1016/j.pt.2011.10.003 22079526

[17] Crump A, Morel CM, Omura S. The onchocerciasis chronicle: from the beginning to the end? Trends Parasitol. 2012; 28: 280–288. doi: 10.1016/j.pt.2012.04.005 22633470

[18] World Health Organization. Framework for the establishment of the Expanded Special Project for Elimination of Neglected Tropical Diseases. World Health Organization Regional Office for Africa. NTD programme. pp.1–24.

[19] Osei-atweneboana MY, Eng J KL, Boakye DA, Gyapong JO, Prichard RK. Prevalence and intensity of Onchocerca volvulus infection and efficacy of ivermectin in endemic communities in Ghana: a two-phase epidemiological study. Lancet. 2007; 369: 2021–2029. doi: 10.1016/S0140-6736(07)60942-8 17574093

[20] Hopkins A. Onchocerciasis then and now: achievements, priorities, and challenges. Community Eye Health. 2017; 30:92–95. 29483756 PMC5820636

[21] Duke BL, Zea-flores G, Castro J and Cupp EW. Effects of multiple monthly doses of ivermectin on adult Onchocerca volvulus. Am J Trop Med Hyg. 1990; 43: 657–664. doi: 10.4269/ajtmh.1990.43.657 2267970

[22] Pion SDS, Nana-Djeunga HC, Kamgno J, Tendongfor N, Wanji S, Njiokou F, et al. Dynamics of Onchocerca volvulus Microfilarial Densities after Ivermectin Treatment in an Ivermectin-naı¨ve and a Multiply Treated Population from Cameroon. PLoS Negl Trop Dis. 2013; 7: e2084. doi: 10.1371/journal.pntd.0002084 23469307 PMC3585010

[23] Awadzi K, Edwards G, Duke BO L, Opoku NO, Attah SK, Addy ET, et al. The co-administration of ivermectin and albendazole—safety, pharmacokinetics, and efficacy against Onchocerca volvulus. Ann Trop Med Parasitol. 2003; 97: 165–178. doi: 10.1179/000349803235001697 12803872

[24] Krotneva SP, Coffeng LE, Noma M, Zouré HGM, Bakoné L, Amazigo UV, et al. African Program for Onchocerciasis Control 1995–2010: Impact of Annual Ivermectin Mass Treatment on Off-Target Infectious Diseases. PLoS Negl Trop Dis. 2015; 9: e0004051. doi: 10.1371/journal.pntd.0004051 26401658 PMC4581698

[25] Freedman DO, Zierdt WS, Lujan A, Nutman TB. The efficacy of ivermectin in the chemotherapy of gastrointestinal helminthiasis in humans. J Infect Dis. 1989; 159: 1151–1153. doi: 10.1093/infdis/159.6.1151 2723457

[26] Naquira C, Jimenez G, Guerra JG, Bernal R, Nalin DR, Neu D, et al. Ivermectin for human strongyloidiasis and other intestinal helminths. Am J Trop Med Hyg. 1989; 40: 304–309. doi: 10.4269/ajtmh.1989.40.304 2929853

[27] Ranque S, Chippaux JP, Garcia A, Boussinesq M. Follow-up of Ascaris lumbricoides and Trichuris trichiura infections in children living in a community treated with ivermectin at 3-monthly intervals. Ann Trop Med Parasitol. 2001; 95: 389–393. doi: 10.1080/00034980120065822 11454248

[28] Heukelbach J, Winter B, Wilcke T, Muehlen M, Albrecht S, Sales de Oliveira FA, et al. Selective mass treatment with ivermectin to control intestinal helminthiases and parasitic skin diseases in a severely affected population. Bull World Health Organ. 2004; 82: 563–571. doi: /S0042-96862004000800005 15375445 PMC2622929

[29] Maegga BTA, Malley KD, Mwiwula V. Impact of ivermectin mass distribution for onchocerciasis control on Ascaris lumbricoides among schoolchildren in Rungwe and Kyela Districts, southwest, Tanzania. Tanzan J Health Res. 2006; 8: 70–74.

[30] Moncayo A, Vaca M, Amorim L, Rodriguez A, Erazo S, Oviedo G, et al. Impact of long-term treatment with ivermectin on the prevalence and intensity of soil-transmitted helminth infections. PLoS Negl Trop Dis. 2008; 2: e293. doi: 10.1371/journal.pntd.0000293 18820741 PMC2553482

[31] Gutman J, Emukah E, Okpala N, Okoro C, Obasi A, Miri ES, et al. Effects of Annual Mass Treatment with Ivermectin for Onchocerciasis on the Prevalence of Intestinal Helminths. Am J Trop Med Hyg. 2010; 83: 534–541. doi: 10.4269/ajtmh.2010.10-0033 20810817 PMC2929048

[32] Oluwole AS, Isiyaku S, Aliero AA, Nwosu C, William A, Elhassan E, et al. Assessment of the burden of soil-transmitted helminthiasis after five years of mass drug administration for Onchocerciasis and Lymphatic filariasis in Kebbi State, Nigeria. Parasite Epidemiol Control. 2017; 2: 21–29. doi: 10.1016/j.parepi.2017.01.002 29774278 PMC5952656

[33] Federal Ministry of Health. Guidelines for onchocerciasis elimination in Ethiopia Addis Ababa, Ethiopia. 2015.

[34] Meribo K, Kebede B, Feleke SM, Mengistu B, Mulugeta A, Sileshi M, et al. Review of Ethiopian onchocerciasis elimination programme. Ethiop Med J. 2017; 55: 55–63. 28878430 PMC5582636

[35] Mengistu G, Balcha F, Britton S. Co-infection of Onchocerca volvulus and intestinal helminths in indigenous and migrant farmers in southwest Ethiopia. Ethiop Med J. 2002; 40: 19–27. 12240564

[36] Taticheff S, Kebede A, Bulto T, Werkeneh W, Tilahun D. Effect of ivermectin (Mectizan) on intestinal nematodes. Ethiop Med J. 1994; 32: 7–15. 8187782

[37] Gebrezgabiher G, Mekonnen Z, Yewhalaw D, Hailu A. Status of parasitological indicators and morbidity burden of onchocerciasis after years of successive implementation of mass distribution of ivermectin in selected communities of Yeki and Asosa districts, Ethiopia. BMC Public Health. 2020; 20: 1–15. doi: 10.1186/s12889-019-7969-5 32787813 PMC7425055

[38] QGIS Development Team (2021). QGIS Geographic Information System. Open Source Geospatial Foundation Project. http://qgis.osgeo.org.

[39] Ritchie LS. An ether sedimentation technique for routine stool examinations. Bull US Army Med Dep. 1948; 8(4):326. 18911509

[40] World Health Organization. Basic Laboratory Methods in Medical Parasitology. World Health Organization. 1991. pp.1–61.

[41] Khurana S, Singh S, Mewara A. Diagnostic techniques for soil-transmitted helminths–Recent advances. Res Rep Trop Med. 2021; 12:181–196. doi: 10.2147/RRTM.S278140 34377048 PMC8349539

[42] Turner HC, Bettis AA, Dunn JC, Whitton JM, Hollingsworth TD, Fleming FM, et al. Economic considerations for moving beyond the Kato-Katz technique for diagnosing intestinal parasites as we move towards elimination. Trends Parasitol. 2017; 33(6):435–43. doi: 10.1016/j.pt.2017.01.007 28187989 PMC5446322

[43] World Health Organization. Prevention and control of schistosomiasis and soil-transmitted helminthiasis: report of a WHO expert committee. World Health Organization technical report series 912. 2002. pp. 1–57. 12592987

[44] Pullan RL, Smith JL, Jasrasaria R, Brooker SJ. Global numbers of infection and disease burden of soil-transmitted helminth infections in 2010. Parasit Vectors. 2014; 7:1–19. doi: 10.1186/1756-3305-7-1 24447578 PMC3905661

[45] World Health Organization. Guideline: preventive chemotherapy to control soil-transmitted helminth infections in at-risk population groups. World Health Organization. 2017. pp.1–75. 29578660

[46] World Health Organization. Helminth control in school-age children: a guide for managers of control programmes. World Health Organization. 2011. pp. 1–75.

[47] Jejaw A, Zemene E, Alemu Y, Mengistie Z. High prevalence of Schistosoma mansoni and other intestinal parasites among elementary school children in Southwest Ethiopia: a cross-sectional study. BMC Public Health. 2015; 15: 1–7. doi: 10.1186/1471-2458-15-1 26135566 PMC4488975

[48] Tekalign E, Bajiro M, Ayana M, Tiruneh A, Belay T. Prevalence and Intensity of Soil-Transmitted Helminth Infection among Rural Community of Southwest Ethiopia: A Community-Based Study. Biomed Res Int. 2019:1–7. doi: 10.1155/2019/3687873 31915688 PMC6931019

[49] Anuar TS, Salleh FM, Moktar N. Soil-Transmitted Helminth Infections and Associated Risk Factors in Three Orang Asli Tribes in Peninsular Malaysia. Sci Rep. 2014; 4: 4101. doi: 10.1038/srep04101 24525479 PMC3924211

[50] Whitworth J, Morgan D, Maude GH, McNichols AM, Taylor DW. A field study of the effects of ivermectin on intestinal helminths in man. Trans R Soc Trop Med Hyg. 1991; 85: 232–234. doi: 10.1016/0035-9203(91)90037-y 1909471

[51] Beach MJ, Streit TG, Addiss DG, Prospere R, Roberts JM, Lammie PJ. Assessment of combined Ivermectin and Albendazole for treatment of Intestinal helminth and *Wuchereria bancrofti* infections in Haitian schoolchildren. Am J Trop Med Hyg. 1999; 60: 479–486. doi: 10.4269/ajtmh.1999.60.479 10466981

[52] Belizario V, Amarillo ME, de Leon WU, de los Reyes AE, Bugayong MG, Macatangay BJ. A comparison of the efficacy of single doses of albendazole, ivermectin, and diethylcarbamazine alone or in combinations against Ascaris and Trichuris spp. Bull World Health Org. 2003; 81: 35–42. 12640474 PMC2572315

[53] Marti H, Haji HJ, Savioli L, Chwaya HM, Mgeni AF, Ameir JS, et al. A comparative trial of a single-dose ivermectin versus three days of albendazole for treatment of *Strongyloides stercoralis* and other soil-transmitted helminth infections in children. Am J Trop Med Hyg. 1996; 55: 477–481. doi: 10.4269/ajtmh.1996.55.477 8940976

[54] Behnke JM, Pritchard DI, Wakelin D, Park JR, McNicholas AM, Gilbert FS. Effect of ivermectin on infection with gastrointestinal nematodes in Sierra Leone. J Helminthol. 1994; 68: 187–195. doi: 10.1017/s0022149x00014334 7829838

[55] Federal Ministry of Health. Tackling Neglected Tropical Diseases through Water, Sanitation, and Hygiene: A national framework to guide integrated programmes in Ethiopia. 2019. Pp.1–21.

