# Supplementary material for: Impact of ivermectin mass drug administration on burden of soil-transmitted helminths in onchocerciasis control and elimination programs, Yeki district, southwest Ethiopia
Source: PLoS One. 2022 Feb 10;17(2):e0263625. doi: 10.1371/journal.pone.0263625 (PMC8830686; doi:10.1371/journal.pone.0263625)
Supplement: S1 Table — (DOCX) [file pone.0263625.s001.docx]

S1 Table: Prevalence of any STH infection by kebele, religion and ethnicity of study participants from Yeki district of southwest Ethiopia (2017)

| **Variable** | | **N examined** | **n positive (%)** |
| --- | --- | --- | --- |
| Kebele | Adisbrehan | 127 | 58(45.7) |
|  | Bechi | 112 | 36(32.1) |
|  | Fide | 48 | 24(50) |
|  | Kubito | 45 | 15(33.3) |
|  | Michi | 47 | 13(27.7) |
|  | Shosha | 51 | 18(35.3) |
|  | Selamber | 38 | 13(34.2) |
|  | Zinki | 23 | 11(47.8) |
| Religion | Protestant | 246 | 108 (43.9) |
|  | Orthodox | 146 | 52(35.6) |
|  | Muslim | 99 | 28(28.3) |
| Ethnicity | Amhara | 178 | 55(30.9) |
|  | Bench | 26 | 13(50) |
|  | Kafficho | 64 | 27(42.2) |
|  | Majang | 86 | 36(41.9) |
|  | Manja | 70 | 29(41.4) |
|  | Oromo | 21 | 7(33.3) |
|  | Shakicho | 10 | 6(60) |
|  | Sheko | 36 | 15(41.7) |
